# Prediction model for myocardial injury after non-cardiac surgery using machine learning

**DOI:** 10.1038/s41598-022-26617-w

**Published:** 2023-01-26

**Authors:** Ah Ran Oh, Jungchan Park, Seo Jeong Shin, Byungjin Choi, Jong-Hwan Lee, Seung-Hwa Lee, Kwangmo Yang

**Affiliations:** 1 Department of Anesthesiology and Pain Medicine, Samsung Medical Center, Sungkyunkwan University School of Medicine, Seoul, Korea; 2 Department of Anesthesiology and Pain Medicine, Kangwon National University Hospital, Chuncheon, Korea; 3 Department of Biomedical Systems Informatics, Yonsei University College of Medicine, Seoul, Korea; 4 Department of Biomedical Sciences, Ajou University Graduate School of Medicine, Suwon, Korea; 5 Heart Vascular Stroke Institute, Rehabilitation & Prevention Center, Samsung Medical Center, Sungkyunkwan University School of Medicine, Seoul, Korea; 6 Department of Biomedical Engineering, Seoul National University College of Medicine, Seoul, Korea; 7 Center for Health Promotion, Samsung Medical Center, Sungkyunkwan University School of Medicine, 81 Irwon-ro, Gangnam-gu, Seoul, Korea; 8 Division of Cardiology, Department of Medicine, Heart Vascular Stroke Institute, Samsung Medical Center, Sungkyunkwan University School of Medicine, 81 Irwon-ro, Gangnam-gu, Seoul, Korea

**Keywords:** Biomarkers, Cardiology, Medical research, Risk factors

## Abstract

Myocardial injury after non-cardiac surgery (MINS) is strongly associated with postoperative outcomes. We developed a prediction model for MINS and have provided it online. Between January 2010 and June 2019, a total of 6811 patients underwent non-cardiac surgery with normal preoperative level of cardiac troponin (cTn). We used machine learning techniques with an extreme gradient boosting algorithm to evaluate the effects of variables on MINS development. We generated two prediction models based on the top 12 and 6 variables. MINS was observed in 1499 (22.0%) patients. The top 12 variables in descending order according to the effects on MINS are preoperative cTn level, intraoperative inotropic drug infusion, operation duration, emergency operation, operation type, age, high-risk surgery, body mass index, chronic kidney disease, coronary artery disease, intraoperative red blood cell transfusion, and current alcoholic use. The prediction models are available at https://sjshin.shinyapps.io/mins_occur_prediction/. The estimated thresholds were 0.47 in 12-variable models and 0.53 in 6-variable models. The areas under the receiver operating characteristic curves are 0.78 (95% confidence interval [CI] 0.77–0.78) and 0.77 (95% CI 0.77–0.78), respectively, with an accuracy of 0.97 for both models. Using machine learning techniques, we demonstrated prediction models for MINS. These models require further verification in other populations.

## Introduction

Myocardial injury after non-cardiac surgery (MINS) is reported to occur in approximately 20% of major surgeries^1^. It is strongly associated with postoperative outcomes, mostly without presenting ischemic symptoms^2^. Therefore, numerous guidelines recommend monitoring perioperative cardiac troponin (cTn) level^3–6^. However, the details of these guidelines are inconsistent, especially in patients needing cTn monitoring^2–6^. Initially, general cardiac risk stratification models in surgical settings were adopted for MINS predictions, and the risk factors in these models have been individually validated for MINS^2,7^. Although previous studies reported the risk factors of MINS^8^, there is no established prediction model, particularly for monitoring of postoperative cTn.

MINS prediction is not a simple task because the mechanism is highly complex, with patient characteristics and operative variables affecting each other^9^. Machine learning has allowed substantial possibilities in medicine, especially in evaluating predictors^10^. The most remarkable advantage of machine learning techniques over traditional statistical models is that they can handle an enormous number of predictors by combining them in nonlinear and highly interactive ways, and this seems suitable for predicting MINS^11^. Therefore, the present study aimed to evaluate MINS predictors and develop prediction models based on machine learning techniques. Using real-world data of consecutive adult patients who underwent non-cardiac surgery with preoperatively normal cTn level, we evaluated the effects of all available variables on postoperative cTn. Based on this result, we eliminated variables to a number suitable for daily clinical practice and generated two prediction models. Our models are available online for verification and for clinicians to adopt them into daily practice.

## Results

### Baseline characteristics and mortality

**Figure 1:**
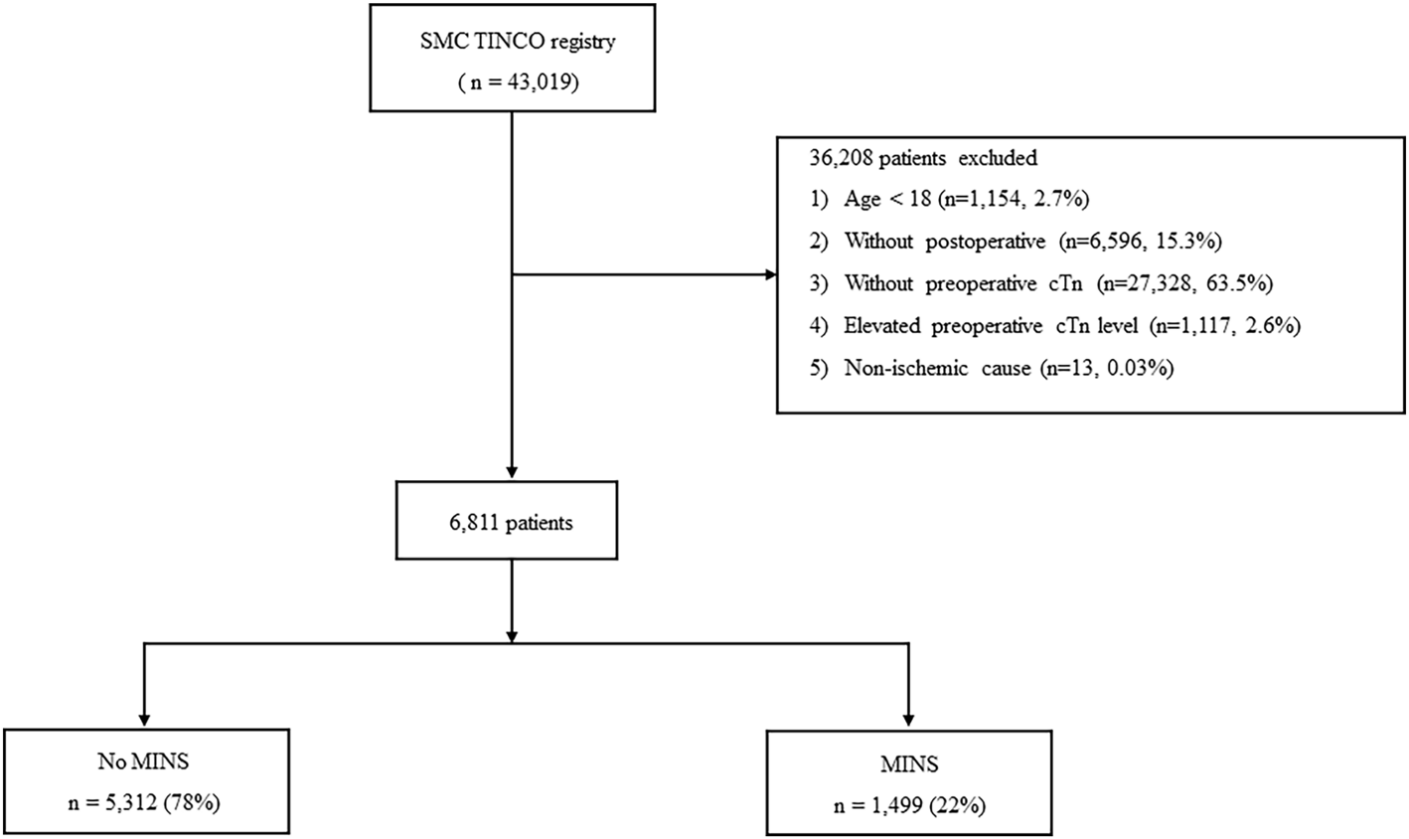
Study patient flowchart.

From the total of 43,019 patients, we excluded (1) 1154 (2.7%) patients younger than 18 years, (2) 6596 (15.3%) patients without postoperative cTn measurement, (3) 27,328 (63.5%) patients without preoperative cTn measurement, (4) 1117 (2.6%) patients with elevated preoperative cTn level, and (5) 13 (0.03%) patients who had a definite non-ischemic cause of postoperative cTn elevation such as pulmonary embolism, sepsis, cardioversion, or atrial fibrillation. Overall, preoperative cTn level was available in 15,195 (35.3%) patients, and 35,882(83.4%) patients had available postoperative cTn levels. The flowchart of the study patients is presented in Fig. 1. In a total of 6811 study patients, MINS was developed in 1499 (22.0%). The baseline characteristics of patients with and without MINS are presented in Table 1. The median value of preoperative cTn level was 6 ng/L (IQR 6–11). The median values of postoperative cTn were 7 ng/L (IQR 6–15) in patients without MINS and 11 ng/L (IQR 6–34) in those with MINS. The median period to peak cTn level was 0.9 days after surgery, and MINS was detected within 48 h after surgery in 77.9% (1168/1499) of MINS patients. The patients with MINS were older, more frequently male, and had higher preoperative cTn level and lower body mass index. They also exhibited higher incidences of most underlying diseases such as heart failure, valvular heart disease, peripheral arterial disease, and chronic pulmonary disease. In contrast, the incidence of active cancer was lower in the MINS group. For preoperative medications, the MINS group was more frequently prescribed beta blocker, calcium channel blocker, angiotensin-converting enzyme inhibitor, antiplatelet agents, and diabetic medications. Operative variables also showed a large difference (Table 1). The patients with MINS more frequently underwent high-risk surgery and emergency operation with a longer duration. Postoperative mortality was higher in patients with MINS (Table 2). The median follow-up period was 2.54 (IQR 1.75–3.42) years.

**Table 1:** Baseline characteristics of patients according to myocardial injury after non-cardiac surgery (MINS).

	No MINS (*N* = 5312)	MINS (*N* = 1499)	*p* value
Preoperative cardiac troponin, ng/L	9.0 (± 6.2)	13.2 (± 9.6)	< 0.001
Male	3174 (59.8)	943 (62.9)	0.03
Age	64.0 (± 12.9)	65.1 (± 14.2)	0.004
Body mass index	24.0 (± 3.8)	23.5 (± 3.9)	< 0.001
Diabetes	2956 (55.6)	889 (59.3)	0.01
Hypertension	3426 (64.5)	1064 (71.0)	< 0.001
Chronic kidney disease	321 (6.0)	205 (13.7)	< 0.001
Dialysis	97 (1.8)	76 (5.1)	< 0.001
Current smoking	692 (13.0)	187 (12.5)	0.6
Current alcohol	1128 (21.2)	237 (15.8)	< 0.001
Coronary artery disease	1016 (19.1)	396 (26.4)	< 0.001
**Previous disease**			
Old myocardial infarction	321 (6.0)	162 (10.8)	< 0.001
History of coronary revascularization	507 (9.5)	218 (14.5)	< 0.001
Percutaneous coronary intervention	444 (8.4)	178 (11.9)	< 0.001
Coronary artery bypass graft	82 (1.5)	54 (3.6)	< 0.001
Heart failure	130 (2.4)	45 (3.0)	0.27
Stroke	504 (9.5)	178 (11.9)	0.01
Atrial fibrillation	362 (6.8)	128 (8.5)	0.03
Arrhythmia	450 (8.5)	157 (10.5)	0.02
Valvular heart disease	94 (1.8)	33 (2.2)	0.33
Aortic disease	142 (2.7)	101 (6.7)	< 0.001
Peripheral arterial disease	202 (3.8)	64 (4.3)	0.45
Chronic pulmonary disease	269 (5.1)	92 (6.1)	0.12
Active cancer	1732 (32.6)	377 (25.2)	< 0.001
**Preoperative treatment**			
Intensive care unit care	371 (7.0)	225 (15.0)	< 0.001
Continuous renal replacement therapy	10 (0.2)	18 (1.2)	< 0.001
Ventilator care	49 (0.9)	38 (2.5)	< 0.001
**Preoperative medication**			
Beta blocker	1422 (26.8)	535 (35.7)	< 0.001
Calcium channel blocker	1857 (35.0)	555 (37.0)	< 0.001
Renin angiotensin aldosterone system inhibitor	2010 (37.8)	563 (37.6)	0.87
Angiotensin-converting enzyme inhibitor	445 (8.4)	171 (11.4)	< 0.001
Angiotensin receptor blocker	1792 (33.7)	488 (32.6)	0.41
Diltiazem	426 (8.0)	128 (8.5)	0.55
Statin	1874 (35.3)	516 (34.4)	0.56
Antiplatelet	2097 (39.5)	650 (43.4)	0.01
Aspirin	1817 (34.2)	522 (34.8)	0.68
Clopidogrel	503 (9.5)	158 (10.5)	0.24
Ticagrelor	277 (5.2)	131 (8.7)	< 0.001
Direct oral anticoagulant	105 (2.0)	27 (1.8)	0.74
Warfarin	318 (6.0)	122 (8.1)	0.003
Diabetic medication	2766 (52.1)	842 (56.2)	0.01
Metformin	849 (16.0)	224 (14.9)	0.35
Insulin	2549 (48.0)	800 (53.4)	< 0.001
**Operative variables**			
General anesthesia	4836 (91.0)	1334 (89.0)	0.02
ESC/ESA surgical high risk	1135 (21.4)	574 (38.3)	< 0.001
Emergency operation	1217 (22.9)	549 (36.6)	< 0.001
Operation duration, hours	3.1 (± 2.2)	4.0 (± 3.1)	< 0.001
**Operation types**			< 0.001
Vascular	934 (17.6)	285 (19.0)	
Orthopedic	563 (10.6)	191 (12.7)	
Neuro	1692 (31.9)	248 (16.5)	
Breast or endo	110 (2.1)	27 (1.8)	
Plastic or otolaryngeal or eye	151 (2.8)	26 (1.7)	
Transplantation	333 (6.3)	316 (21.1)	
Gynecology or urology	168 (3.2)	52 (3.5)	
Gastrointestinal	1092 (20.6)	234 (15.6)	
Noncardiac thoracic	256 (4.8)	114 (7.6)	
Others	13 (0.2)	6 (0.4)	
**Intraoperative treatment**			
Red blood cell transfusion	502 (9.5)	357 (23.8)	< 0.001
Inotropic drug infusion	1897 (35.7)	812 (54.2)	< 0.001

Data are presented as n (%), mean (± standard deviation).

ESC, European Society of Cardiology; ESA, European Society of Anaesthesiology.

**Table 2:** Mortalities according to myocardial injury after non-cardiac surgery (MINS).

	No MINS (*N* = 5312)	MINS (*N* = 1499)	*p* value
Overall mortality	969 (18.2)	449 (30.0)	< 0.001
Cardiovascular mortality	394 (7.4)	156 (10.4)	< 0.001
One-year mortality	489 (9.2)	309 (20.6)	< 0.001
Cardiovascular mortality	147 (2.8)	90 (6.0)	< 0.001
30-day mortality	73 (1.4)	126 (8.4)	< 0.001
Cardiovascular mortality	15 (0.3)	29 (1.9)	< 0.001

Data are presented as n (%).

### A predictive model for MINS

**Figure 2:**
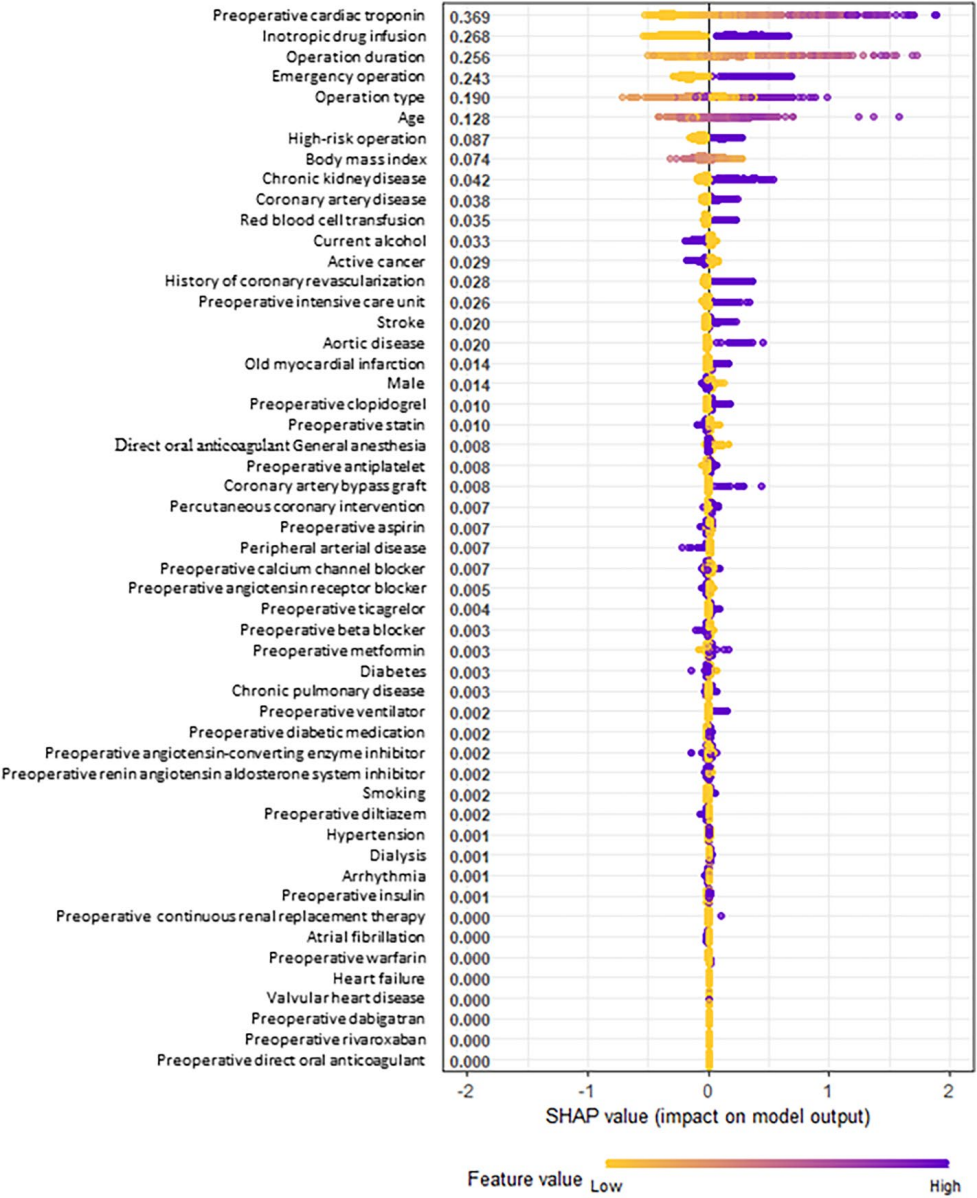
SHapley additive exPlanations (SHAP) summary plot representing the results of the extreme gradient boosting (XGB) algorithm of machine learning techniques.

**Figure 3:**
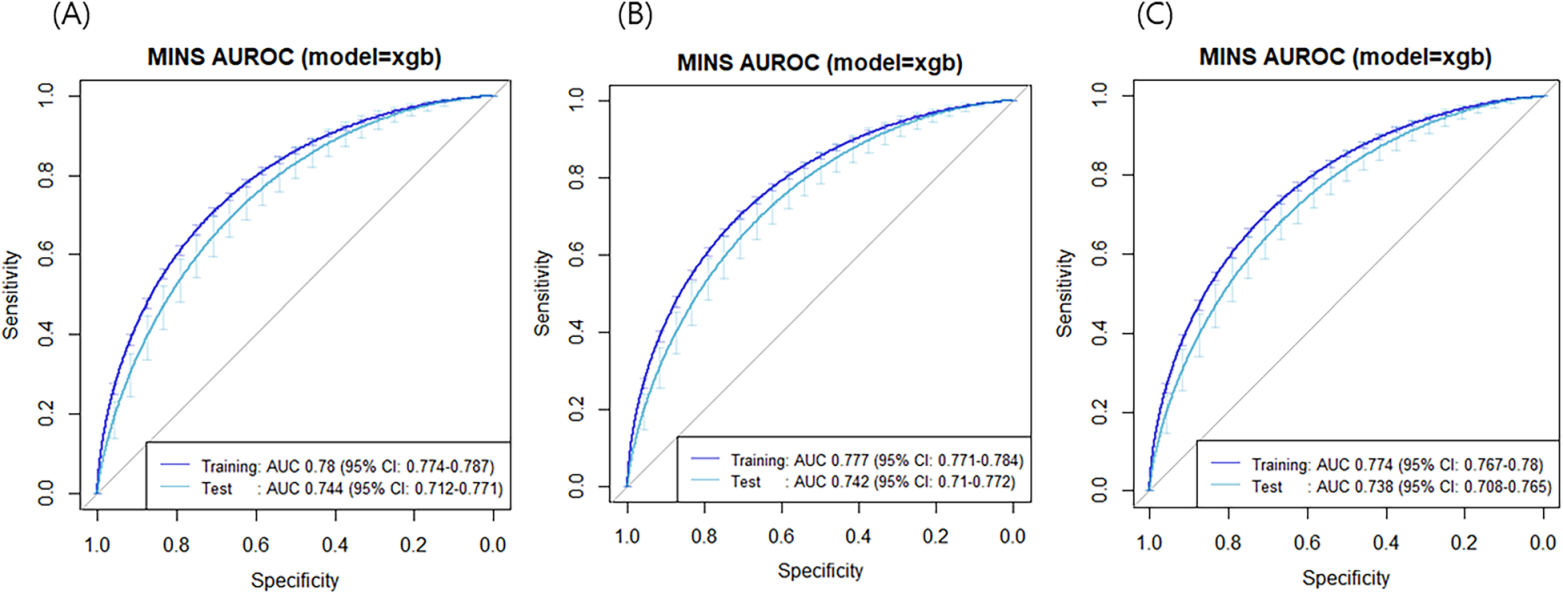
The receiver operating characteristic curves of the (**A**) 52-variable model, (**B**) 12-variable model, and (**C**) 6-variable model.

The SHAP summary plot for the results of the XGB model is shown in Fig. 2. The input variables, as listed on the y-axis, are ranked from most important (top) to least important (bottom) according to their contributions to the development of MINS. The x-axis presents the influence of variables on the prediction of MINS. A positive SHAP value indicates that the feature value increases the likelihood of MINS, and a negative SHAP value is associated with lower risk. The top 12 variables were preoperative cTn level (0.369), intraoperative inotropic drug infusion (0.268), operation duration (0.256), emergency operation (0.243), operation type (0.190), age (0.128), high-risk surgery (0.087), body mass index (0.074), chronic kidney disease (0.042), coronary artery disease (0.038), intraoperative red blood cell transfusion (0.035), and current alcoholic use (0.033). The prediction model using all 52 variables showed an AUROC of 0.78 (95% CI 0.77–0.79) and accuracy, sensitivity, and specificity of 0.81, 0.37, and 0.93, respectively (Fig. 3).

For practical use in daily practice, we eliminated variables according to SHAP value. We developed two prediction models by retaining the top 12 variables with SHAP value > 0.03 and the top 6 variables with SHAP value > 0.1. These models are available at https://sjshin.shinyapps.io/mins_occur_prediction/ (Supplementary Fig. S1). When values for the variables of the target patient were entered, the probability for MINS is shown as an output. The estimated thresholds were 0.47 for the 12-variable model and 0.53 for the 6-variable model. The receiver operating characteristic curves of the models are shown in Fig. 3. The prediction models exhibited an AUROC of 0.78 (95% CI 0.77–0.78) for the 12-variable model and 0.77 (95% CI 0.77–0.78) for the 6-variable model. Accuracy, sensitivity, and specificity were 0.79, 0.29, and 0.93 in the 12-variable model and 0.79, 0.21, and 0.96 in the 6-variable model, respectively (Fig. 3).

## Discussion

In this study, we used machine learning techniques with an XGB algorithm to identify variables associated with MINS and created prediction models. The incidence of MINS, defined by cTn elevation above the upper reference limit, in patients with preoperatively normal cTn level was 22.0%. The top 12 variables retained in our prediction models were preoperative cTn level, intraoperative inotropic drug infusion, operation duration, emergency operation, operation type, age, high-risk surgery, body mass index, chronic kidney disease, coronary artery disease, intraoperative red blood cell transfusion, and current alcoholic use. We created two models according to number of variables, and the prediction models achieved an AUROC of 0.78 (95% CI 0.77–0.78) for the 12-variable model and 0.77 (95% CI 0.77–0.78) for the 6-variable model.

Current guidelines recommend selective monitoring of postoperative cTn, but there are still difficulties in predicting the probability of MINS^2–6^. In this study, we included patients who had available pre- and postoperative cTn level to exclude patients with chronic cTn elevation. Two discrete mechanisms are involved in development of MINS. Although oxygen supply–demand mismatch outnumbers thrombosis, risk factors for both mechanisms should be considered in MINS development^12^. In addition, non-ischemic causes that contribute to cTn elevation are frequently found in the perioperative period, complicating prediction of MINS^13^. Machine learning might be a suitable tool to interpret interactive data from electronic hospital records and transform them into knowledge^10^. In this study, we curated real-world data directly from the electronic hospital records of consecutive patients undergoing non-cardiac surgery with preoperatively normal cTn level and investigated the effects of variables on postoperative cTn elevation. We applied machine learning techniques with the XGB algorithm, known as the best performing algorithm^14^. In our previous study, we compared performances of various machine learning algorithms for prediction of patients with mortality after MINS, and XGB was shown to be the best performing algorithm^15^.

One of the issues in interpreting results of the machine learning techniques is that causal inference of observational data is not resolved^16^. In other words, predictors from machine learning techniques are not necessarily causes of an event^16^. However, variables that were selected for our predictive model exhibited clinical relevance. According to our result, preoperative cTn showed the largest effect on MINS, despite our inclusion of only patients with preoperative cTn level within normal range. In the perioperative period, cTn level even within the normal range was reported to be associated with outcome^17^. The current guidelines do not provide a clear recommendation for preoperative cTn measurement^2–6^, and only the guideline from Canadian society refers to the need for baseline cTn level^3^. Our model supprots that preoperative cTn level may need to be measured in high-risk patients. Numerous variables in our model reflected myocardial burden from surgical procedures such as intraoperative inotropic drug use, emergency operation or duration of the procedure. The need for intraoperative inotropic drug infusion and red blood cell transfusion also might be related to hypotension or anemia, which is associated with a higher risk of MINS^18–20^. In addition, transfusions per se could act as an additional burden^21,22^. On the other hand, this may also be due to pre-existing anemia, and this needs further investigation. Regarding the types of surgery, there was no case where intraoperative cardiopulmonary bypass was required. A higher incidence of MINS was reported in thoracic surgery where the pericardium was manipulated based on the extent of lung resection^23^, and a similar result was observed in our model.

Our models also retained known risk factors from patient characteristics such as age and previous history of cardiovascular disease. Postoperative monitoring of cTn was recommended for patients over 45 years of age as an expert opinion^24^, and the cost to monitor MINS was appealing per health gain for patients over 65 years of age^25^. The association with body mass index was also reported. Although obese individuals are known to have higher risks of cardiovascular disease and death, the “obesity paradox” of lower mortality in mildly obese patients has been suggested for MINS and perioperative myocardial injury^26,27^.

The strength of our models is the feasibility to be adopted into daily clinical practice after further validation, because the variables are clinically convincing and readily available from routine medical records. For user convenience, we provided multiple models based on less number of retained variables and showed similar predictive values. We also provided the estimated cut-off values of each model according to our dataset. However, whether the model with more variables could offer superior predictive value and the optimal cut-off value that can be universally applied needs further validation. In addition, the low sensitivity of the model limits the use as a screening test in a clinical practice. It seems more reasonable to consider this model when ruling out low-risk patients rather than to select high-risk patients, considering the high specificity and low sensitivity. This could help sparing a limited medical resources from patients who were ruled out from MINS. In this model, we only included preoperative variables, so it could be used from the preoperative period when applied into the clinical practice. Some of our variables were even modifiable, but it is unclear whether modification of these variables could reduce the incidence of MINS. An effective method to prevent MINS has yet to be established^2,7^, and sparing a limited resource from low-risk patients based on our model could be a good start for an early identification and treatment of MINS patients. However, in this study, we evaluated various preoperative medications, but none exhibited a meaningful effect on MINS occurrence. This is in line with previous findings where the use of beta blockers decreased postoperative myocardial infarction but increased the incidence of stroke^28^. Other cardiovascular drugs including aspirin, nitrous oxide, and clonidine in the preoperative period exhibited non-significant results for MINS prevention^7^.

Our study has several limitations that must be considered. First, this study used single-center retrospective data, and there is a residual risk of confounding effects of unmeasured factors. Our analysis lacked detailed cardiac evaluations such as echocardiography since not all patients had such data. Preoperative results of other blood laboratory tests and intraoperative variables that could not be retained owing to the lack of data availability may need to be taken into account in future studies. To exclude patients with chronic cTn elevation, we enrolled those with available preoperative cTn level, and numerous patients were excluded due to the absence of preoperative cTn level. Moreover, perioperative cTn was selectively measured, so the incidence of MINS might have been overestimated, and there may be patients who were supposed to be evaluated with cTn but were not. Furthermore, postoperative cTn was not monitored systemically. There may be patients who were lost during cTn monitoring, and a graded association could not be evaluated. In addition, our study was conducted among cTn I, and the results might have differed according to the cTn assay. So, for our model to become generalizable, it needs further internal and external validations, especially in patients where cTn was routinely measured. In addition, the definition of non-ischemic cause of cTn elevation was strictly applied owing to the retrospective nature of the study, and this may have caused selection bias. In further study, different models may need to be developed according to types of surgery and emergency procedures. Additionally, our study population showed relatively high mortality, because they were high-risk patients in whom cTn was measured in both pre- and postoperative periods. This may have also caused selection bias. Lastly, perioperative management was not well-controlled. Although we followed the institutional protocol based on current guidelines, this might have been updated during the study period. Despite these limitations, this is the first study to demonstrate predictive models of MINS based on risk factors identified by machine learning techniques.

## Conclusion

Based on the results of machine learning techniques, we demonstrated prediction models of MINS, which we made available online. These models require further verification among other populations.

## Methods

### Ethics

The Institutional Review Board of Samsung Medical Center approved this study and the requirement for informed consent was waived because this study used retrospectively collected de-identified data (Samsung Medical Center, 81 Irwon-ro, Gangnam-gu, Seoul, Korea, 2021-06-078). Our study was conducted following the principles outlined in the Declaration of Helsinki, and the results were reported following the “Strengthening the Reporting of Observational Studies in Epidemiology” guidelines.

### Study population and data curation

WE created the Samsung Medical Center-Troponin In NonCardiac Operation (SMC-TINCO) registry (KCT0004244), which is a single-center de-identified cohort of 43,019 consecutive patients who underwent non-cardiac surgery with at least one measurement of cTn value during 30 days before or after the surgery in Samsung Medical Center, Seoul, Korea, between January 2010 and June 2019. Raw data for the registry were extracted from our institutional electronic archive system which contains electronic hospital records of over 4 million patients with more than 900 million laboratory findings and 200 million prescriptions. We used “Clinical Data Warehouse Darwin-C,” an electronic system that allows investigators to search and retrieve de-identified medical records. To assess the mortality data outside our institution, this system is consistently renewed and verified with the National Population Registry of the Korea National Statistical Office using a unique personal identification number. The blinded investigators collected related preoperative variables such as demographic data, underlying disease, and blood laboratory tests from the preoperative evaluation sheet based on medical information extracted from electronic hospital records where the patients self-reported their comorbidities We also adapted International Classification of Diseases-10 codes to curate missed underlying disease and calculate the Charlson comorbidity index based on the preoperative diagnosis^29^.

Postoperative events were investigated based on the extracted in-hospital progress notes, nursing charts, discharge notes, results of examinations, and drug prescriptions. All patients in this registry completed 30 days of follow-up to detect MINS and mortality.

From the entire registry, we excluded the following patients: (1) patients younger than 18 years, (2) patients without preoperative or postoperative cTn data, (3) patients with elevated preoperative cTn level, and (4) patients who had a definite non-ischemic cause of cTn elevation such as pulmonary embolism, sepsis, cardioversion, or atrial fibrillation. After finalizing 6811 patients for this study, they were divided into two groups according to MINS occurrence.

### Study endpoint and definitions

The primary endpoint was MINS, and we aimed to demonstrate a prediction model for MINS using machine learning techniques. We quantified and compared the effect of each variable on the predictive performance of the models. After conducting feature elimination, we developed a calculator for MINS prediction and provided it online.

Following the current diagnostic criteria, MINS was defined as a peak cTn level above the 99th percentile of the upper reference limit within 30 days after surgery^2,7^. Elevation of cTn with a definite non-ischemic cause such as pulmonary embolism, sepsis, cardioversion, chronic elevation, or atrial fibrillation was not regarded as MINS. Active cancer was defined as a histologic confirmation of malignancy within six months before surgery^30^. High-risk surgical procedures were selected following the European Society of Cardiology (ESC)/European Society of Anaesthesiology (ESA) guidelines on non-cardiac surgery^5^.

### Perioperative cTn measurement and management

According to our institutional protocol, perioperative cTn was measured in patients undergoing moderate to high-risk surgery or in those undergoing low-risk surgery with at least one major cardiovascular risk factor such as a history of ischemic heart disease, heart failure, stroke including transient ischemic attack, diabetes mellitus on insulin therapy, or chronic kidney disease based on current guidelines^5^. In patients with minor risk factors, attending clinicians performed cTn measurement at their own discretion based on the patient’s recent symptoms suspected of ischemic heart disease or advanced age. An automated analyzer (Advia Centaur XP; Siemens Healthcare Diagnostics, Erlangen, Germany) with a highly sensitive cTn I immunoassay was used. According to the manufacturer, the lowest limit of detection was 6 ng/L, and the 99th percentile URL was 40 ng/L^31^. Patients with elevated cTn were referred to a cardiologist for consultation and were managed appropriately by an attending clinician.

### Development of prediction models

We used a machine learning technique with an extreme gradient boosting (XGB) algorithm provided by the xgboost package of R. It is a boosting ensemble prediction model based on decision trees implementing machine learning algorithms under the Gradient Boosting framework^14,32^. Optimization of hyper-parameters was based on grid searches using the area under the receiver operating characteristic (AUROC), and five-fold cross-validation was implemented during model development. We conducted a stratified random split of the data with a constant ratio of patients with MINS occurrence to divide the data into training and testing sets. Of the data, 80% was used for training the machine learning model, and the remaining 20% was for the testing model.

For model interpretation, feature importance on MINS was reported based on SHapley Additive exPlanations (SHAP) values and presented in the SHAP summary plot. The SHAP value explains the intensity and direction of impact on the outcome of interest and is determined by comparing the prediction of the model with and without the feature^32^. In the SHAP summary plot, features are arranged in descending order by the effect on the outcome of interest, and one dot on each variable line represents each patient. The x-axis depicts the direction and magnitude of the impact. Features with positive SHAP values suggest directly proportional variables to the outcome of interest, and those with negative SHAP values suggest an inverse correlation.

For easy access to the prediction model in clinical practice, we eliminated the features using Recursive Feature Elimination with cross validation. In this method, we eliminated features starting from those with less importance while observing the performance of the models. We developed prediction models for MINS with less number of variables using leveraging R Shiny. Users can develop the application freely via public link. We demonstrated two prediction models using the features with the top 12 and 6 SHAP values. An optimal threshold for probability was estimated using Youden's J statistic, and AUROC, accuracy, sensitivity, and specificity were also provided.

### Statistical analysis

We compared the differences between patients who developed MINS and those who did not. Continuous features were expressed as mean ± standard deviation or median with interquartile range (IQR). The Student's t-test was used for comparisons between parametric data, and nonparametric data were analyzed with the Mann–Whitney U test. Categorical variables were presented as a number with percentage, and chi-square or Fisher's exact tests were used to test for differences between groups as appropriate. All statistical analysis was performed with R 4.1.2 (Vienna, Austria; http://www.R-project.org/).

### Supplementary Information

Supplementary Figure S1.

## Data Availability

The datasets generated during and/or analysed during the current study are available from the corresponding author on reasonable request.

## References

[1] Bartels K, Karhausen J, Clambey ET, Grenz A, Eltzschig HK (2013). Perioperative organ injury. Anesthesiology.

[2] Ruetzler K (2021). Diagnosis and management of patients with myocardial injury after noncardiac surgery: A scientific statement from the american heart association. Circulation.

[3] Duceppe E (2017). Canadian cardiovascular society guidelines on perioperative cardiac risk assessment and management for patients who undergo noncardiac surgery. Can. J. Cardiol..

[4] Fleisher LA (2014). 2014 ACC/AHA guideline on perioperative cardiovascular evaluation and management of patients undergoing noncardiac surgery: A report of the American College of Cardiology/American Heart Association Task Force on practice guidelines. J. Am. Coll. Cardiol..

[5] Kristensen SD, Knuuti J (2014). New ESC/ESA Guidelines on non-cardiac surgery: Cardiovascular assessment and management. Eur. Heart J..

[6] De Hert S (2018). Pre-operative evaluation of adults undergoing elective noncardiac surgery: Updated guideline from the European Society of Anaesthesiology. Eur. J. Anaesthesiol..

[7] Devereaux PJ, Szczeklik W (2020). Myocardial injury after non-cardiac surgery: Diagnosis and management. Eur. Heart J..

[8] Weersink CSA, van Waes JAR, Grobben RB, Nathoe HM, van Klei WA (2021). Patient selection for routine troponin monitoring after noncardiac surgery. J. Am. Heart Assoc..

[9] Puelacher C (2020). Etiology of peri-operative myocardial infarction/injury after noncardiac surgery and associated outcome. J. Am. Coll. Cardiol..

[10] Obermeyer Z, Emanuel EJ (2016). Predicting the future—big data, machine learning, and clinical medicine. N. Engl. J. Med..

[11] Mullainathan S, Spiess J (2017). Machine learning: An applied econometric approach. J. Econ. Perspect..

[12] Sheth T (2018). Incidence of thrombosis in perioperative and non-operative myocardial infarction. Br. J. Anaesth..

[13] Devereaux PJ (2017). Association of postoperative high-sensitivity troponin levels with myocardial injury and 30-day mortality among patients undergoing noncardiac surgery. JAMA.

[14] Association for Computing Machinery Special Interest Group on Management of Data & ACM Special Interest Group on Knowledge Discovery in Data. In *Proceedings of the 22nd ACM SIGKDD International Conference on Knowledge Discovery and Data Mining* 785–794 (ACM, 2016).

[15] Shin SJ, Park J, Lee SH, Yang K, Park RW (2021). Predictability of mortality in patients with myocardial injury after noncardiac surgery based on perioperative factors via machine learning: Retrospective study. JMIR Med. Inform..

[16] Kleinberg J, Ludwig J, Mullainathan S, Obermeyer Z (2015). Prediction policy problems. Am. Econ. Rev..

[17] Park J (2020). Preoperative cardiac troponin below the 99th-percentile upper reference limit and 30-day mortality after noncardiac surgery. Sci. Rep..

[18] Turan A (2021). Association between postoperative haemoglobin and myocardial injury after noncardiac surgery: A retrospective cohort analysis. Br. J. Anaesth..

[19] Kwon JH (2021). Pre-operative anaemia and myocardial injury after noncardiac surgery: A retrospective study. Eur. J. Anaesthesiol..

[20] Salmasi V (2017). Relationship between intraoperative hypotension, defined by either reduction from baseline or absolute thresholds, and acute kidney and myocardial injury after noncardiac surgery: A retrospective cohort analysis. Anesthesiology.

[21] Gillies MA (2021). A restrictive versus liberal transfusion strategy to prevent myocardial injury in patients undergoing surgery for fractured neck of femur: A feasibility randomised trial (RESULT-NOF). Br. J. Anaesth..

[22] Park J (2021). Intraoperative blood loss may be associated with myocardial injury after non-cardiac surgery. PLoS ONE.

[23] Gonzalez-Tallada A (2020). Myocardial injury after noncardiac surgery: Incidence, predictive factors, and outcome in high-risk patients undergoing thoracic surgery: An observational study. J. Cardiothorac. Vasc. Anesth..

[24] Sessler DI, Devereaux PJ (2016). Perioperative troponin screening. Anesth. Analg..

[25] Buse GL (2018). Troponin T monitoring to detect myocardial injury after noncardiac surgery: A cost-consequence analysis. Can. J. Surg..

[26] Lee SH (2021). Association between high body mass index and mortality following myocardial injury after noncardiac surgery. Anesth. Analg..

[27] Hidvegi R (2020). Obesity paradox and perioperative myocardial infarction/injury in non-cardiac surgery. Clin. Res. Cardiol..

[28] Devereaux PJ (2008). Effects of extended-release metoprolol succinate in patients undergoing non-cardiac surgery (POISE trial): A randomised controlled trial. Lancet.

[29] Sundararajan V (2004). New ICD-10 version of the Charlson comorbidity index predicted in-hospital mortality. J. Clin. Epidemiol..

[30] Lee AYY (2015). Tinzaparin vs warfarin for treatment of acute venous thromboembolism in patients with active cancer: A randomized clinical trial. JAMA.

[31] Mahajan VS, Jarolim P (2011). How to interpret elevated cardiac troponin levels. Circulation.

[32] Aas K, Jullum M, Løland A (2021). Explaining individual predictions when features are dependent: More accurate approximations to Shapley values. Artif. Intell..

