# Author Correction: Prediction model for myocardial injury after non-cardiac surgery using machine learning

**DOI:** 10.1038/s41598-023-43067-0

**Published:** 2023-09-22

**Authors:** Ah Ran Oh, Jungchan Park, Seo Jeong Shin, Byungjin Choi, Jong‑Hwan Lee, Seung‑Hwa Lee, Kwangmo Yang

**Affiliations:** 1 Department of Anesthesiology and Pain Medicine, Samsung Medical Center, Sungkyunkwan University School of Medicine, Seoul, Korea; 2 Department of Anesthesiology and Pain Medicine, Kangwon National University Hospital, Chuncheon, Korea; 3 Department of Biomedical Systems Informatics, Yonsei University College of Medicine, Seoul, Korea; 4 Department of Biomedical Sciences, Ajou University Graduate School of Medicine, Suwon, Korea; 5 Heart Vascular Stroke Institute, Rehabilitation & Prevention Center, Samsung Medical Center, Sungkyunkwan University, School of Medicine, Seoul, Korea; 6 Department of Biomedical Engineering, Seoul National University College of Medicine, Seoul, Korea; 7 Center for Health Promotion, Samsung Medical Center, Sungkyunkwan University School of Medicine, 81 Irwon‑Ro, Gangnam‑Gu, Seoul, Korea; 8 Division of Cardiology, Department of Medicine, Heart Vascular Stroke Institute, Samsung Medical Center, Sungkyunkwan University School of Medicine, 81 Irwon‑Ro, Gangnam‑Gu, Seoul, Korea

Correction to: *Scientific Reports* 10.1038/s41598-022-26617-w, published online 26 January 2023

The original version of this Article contained an error in the Funding section.

“This work was funded by Ministry of Health and Welfare (Grant no. NIPA 1711120339).”

now reads:

“This work was funded by Ministry of Science and ICT (Grant no. NIPA 1711120339).”

The original Article has been corrected.

