# Supplementary figures and images for: Prediction model for myocardial injury after non-cardiac surgery using machine learning
Source: Sci Rep. 2023 Jan 26;13:1475. doi: 10.1038/s41598-022-26617-w (PMC9879966; doi:10.1038/s41598-022-26617-w)

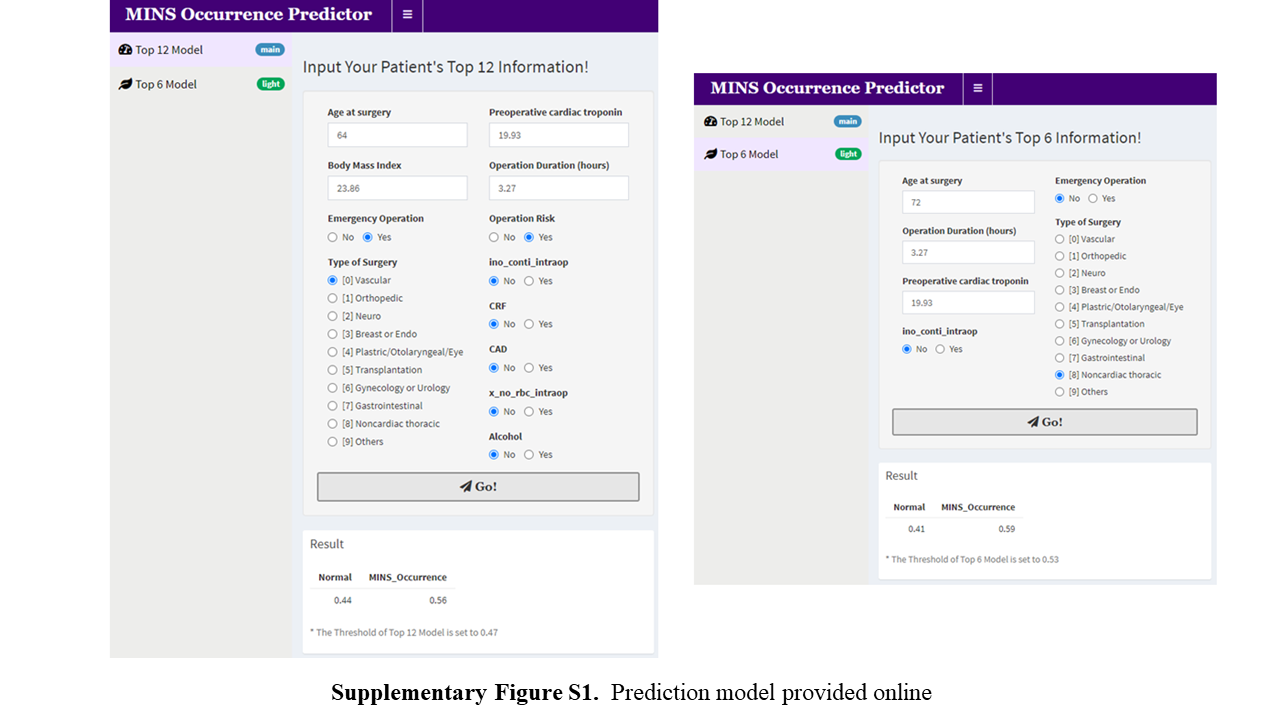

Supplement: Supplementary file 1 — Supplementary Figure S1. [file 41598_2022_26617_MOESM1_ESM.tif]
